# Implementing mHealth Apps Through Community Engagement to Promote Cancer Screening: A Scoping Review

**DOI:** 10.3390/healthcare13101161

**Published:** 2025-05-16

**Authors:** Maria Teresa Riccardi, Aurora Heidar Alizadeh, Bianca Maria Costigliolo, Anna Nisticò, Lia Olivo, Mario Cesare Nurchis, Massimo Maurici, Elisabetta Anna Graps, Massimo Oddone Trinito, Gianfranco Damiani

**Affiliations:** 1 Local Health Unit Roma 2, 00159 Rome, Italy; mt.riccardi@gmail.com (M.T.R.); massimo.trinito@aslroma2.it (M.O.T.); 2 Department of Biomedicine and Prevention, University of Rome “Tor Vergata”, 00133 Rome, Italy; maurici@med.uniroma2.it; 3 Department of Health Science and Public Health, Section of Hygiene, Università Cattolica del Sacro Cuore, 00168 Rome, Italy; aurora.heidar@gmail.com (A.H.A.); annanisti@gmail.com (A.N.); lia.olivo01@icatt.it (L.O.); gianfranco.damiani@unicatt.it (G.D.); 4 Faculty of Medicine and Surgery, Università Cattolica del Sacro Cuore, 00168 Rome, Italy; biancamaria.costigliolo01@icatt.it; 5 Department of Life Science, Health, and Health Professions, Università Degli Studi Link, 00165 Rome, Italy; 6 ARESS Puglia—Agenzia Regionale Strategica per la Salute ed il Sociale, Presidenza della Regione Puglia, 70121 Bari, Italy; e.graps@aress.regione.puglia.it

**Keywords:** cancer screening, mHealth, community engagement

## Abstract

**Background/Objectives**: Colorectal (CRC), breast (BC), and cervical cancer (CC) pose a significant health burden, yet screening programs have been proven to reduce cancer-specific mortality and other non-lethal endpoints. Mobile health (mHealth) technologies can enhance adherence, but effectiveness varies. This scoping review aims to explore mHealth apps for cancer screening developed with community engagement, identifying research approaches and gaps. **Methods**: A scoping review following PRISMA-ScR guidelines analyzed studies on mHealth apps for cancer screening developed through community engagement. Community engagement was classified per WHO’s definition. Databases were searched using a PCC-based strategy; eligible studies involved app development, excluding hypothetical apps or text messaging-/social media-only interventions. Screening and data extraction were conducted independently. **Results**: Thirteen articles were included. Findings indicate a growing but limited body of evidence, with most studies focusing on CRC and BC and involving minority populations through mHealth apps. Key engagement phases included research design, CAB establishment, and recruitment, while priority setting was never community-led. The wMammogram, Meet ALEX, and mMammogram apps improved screening knowledge, intention, and participation, while ColorApp enhanced knowledge but not attitudes. Only CBPR-based studies included dissemination, and one involved the CAB in data analysis. Some studies acknowledged community contributions, though details on ColorApp’s engagement were limited. **Conclusions**: Standardized engagement frameworks combined with mHealth were associated with greater community involvement and may improve equity. No community-designed mHealth app was found for CC screening, despite its relevance. Future research should address gaps in CC programs, prioritize early community involvement, and assess the long-term impact of mHealth interventions.

## 1. Introduction

In 2022, global cancer incidence exceeded 19 million, contributing nearly 10 million deaths to global mortality. Colorectal cancer (CRC) is the second leading cause of cancer-related deaths worldwide, with more than 1.9 million new cases in 2022. In women, breast cancer (BC) is the most common cancer, with more than 2 million cases. Data on cervical cancer (CC) report almost 350,000 deaths and more than 600,000 new cases in 2022 [1]; according to another 2022 study on global burden of CC, the age-standardized mortality rate was 7.08 for 100,000 women, thus representing the fourth cause of cancer morbidity and mortality in women worldwide. Peculiarly, preventive strategies for CC include a vaccine against HPV, unlike other types of cancer screening; in fact, CC could be ended as a public health problem through widespread implementation of the WHO initiative to eliminate cervical cancer [2,3].

Empirical evidence has shown that systematic oncological screening, along with appropriate preventive measures (i.e., lifestyle changes), significantly reduces the mortality associated with certain cancers [4] and has a worthwhile impact on non-lethal endpoints (e.g., aggressiveness of treatment, costs) [5] and on other relevant patient-related outcomes (e.g., quality of life, satisfaction) [6]. As a result, many health authorities in different countries have implemented population-level screening initiatives as a preventive public health measure [7] addressing CC, BC, and CRC, following specific guidelines and periodical revisions of scientific evidence [8]. As recommended by the WHO, indeed, the types of cancer that should undergo screening, in the eligible target population, are currently CC, BC, and CRC [9]. Additional programs for other cancer types are being planned and assessed (e.g., lung and prostate cancer screening) [10,11].

The current communication technologies available, like social media, facilitate personal and community health literacy and education, disease surveillance, and mass information on public health matters [12]; since existing screening methods make CC, BC, and CRC preventable, it is even more important to increase public awareness of the practices of cancer prevention and early detection [13].

Mobile health (mHealth), a subset of electronic health (eHealth), refers to the application of mobile communication technologies to support healthcare processes [14]. The rising adoption of mHealth is driven not only by its established advantages, but also by the broad penetration of mobile phones and the ease of use they offer, even for individuals with limited literacy [15]. Indeed, the integration of mobile health (mHealth) technologies in healthcare enables more frequent communication, the provision of personalized content, and enhanced accessibility to health-related information [16].

mHealth interventions have been studied in a variety of health domains, including weight loss [17], behavior change [18,19], cancer screening [7], and mental wellness [20], and also in low- and middle-income countries where access to the internet and mobile phones has been shown to be comparable to that in high-income countries [13].

Research on digital health interventions highlights the need for comprehensive planning to ensure seamless integration into healthcare systems and facilitate patient use [21]. The scientific literature shows that not all mHealth interventions can be effective in increasing screening adherence, so community involvement at all stages of implementation can be a key element [22]. Co-design, for example, helps uncover the critical factors influencing a program or practice within a given context while also identifying facilitators and barriers to the subsequent implementation phase [23].

Despite a growing body of evidence, there are few projects documented in the scientific literature in which mHealth apps to promote adherence to cancer screening are co-designed with the population through community engagement mechanisms.

The aim of this study was to explore the features and characteristics of those mobile apps designed to promote cancer screening that were implemented through a community engagement process, in order to examine how research is conducted in this area and to identify potential gaps [24].

## 2. Materials and Methods

A scoping review of the literature was conducted to review mHealth Apps implemented through a community engagement process to promote cancer screening, following the Preferred Reporting Items for Systematic Reviews and Meta-Analyses extension for Scoping Reviews (PRISMA-ScR) [25], a methodological framework developed by Tricco et al. and informed by the Joanna Briggs Institute [26]. The scoping review protocol was registered on OSF (registration code: 10.17605/OSF.IO/GSR64).

### 2.1. Search Strategy

Medline, ISI Web of Science, and Scopus databases were queried without any data or language restriction. A search string was created according to the PCC (Population, Concept and Context) framework, using the following terms: “cervical cancer screening”, “breast cancer screening”, “colorectal cancer screening”, “community engagement”, “mHealth”, and “mobile app”. Potentially missing articles were obtained through snowball searching. Databases were queried until the end of February 2025.

### 2.2. Study Selection

Only studies that developed an app through community engagement were included. We excluded studies that only surveyed user preferences about hypothetical apps without proceeding to actual app development or implementation. To ensure the inclusion of as many studies as possible, of the qualitative articles that investigated preferences via community engagement, we manually searched subsequent articles by the same authors regarding the implementation and use of an app including the retrieved studies. Studies including mHealth that were limited to text messaging or social media were excluded. The outcomes considered were as follows: screening adherence, defined as the proportion of adults who participated in the screening; app features modified by or co-designed using community engagement methods; and engagement of population, defined as the intent to participate in a screening program within a specified timeframe.

The first round of article screening assessing the title and abstract was conducted by two independent researchers (BC and AHA). Then, the full texts of the included studies were screened to determine the final eligibility by three independent researchers (BC, AHA, and AN). If disagreements arose, a fourth author (MTR) would be consulted.

A quality assessment of the included studies was not performed, since the study design does not require it [26].

### 2.3. Data Extraction

Two reviewers (MTR and BC) independently extracted data from the included studies using a piloted data collection form in Excel (Version 15.0; Microsoft). Any discrepancies were resolved by discussion. The extracted information included study characteristics (authors, date of publication, country, type of publication and study design, screening program, community engagement framework, and phases involving population), participant characteristics (eligibility criteria), app features, outcomes of interest, and measurement tools.

### 2.4. Data Synthesis

The community engagement definition followed was the one by WHO, stating that it is a process of building connections that empower stakeholders to collaborate in addressing health-related challenges and promoting well-being to achieve positive health outcomes and impact, and it could vary in level, depth, and scope, influencing the type and extent of people’s involvement [27]. The phases involving the participation of the community of interest were extracted and classified into the following categories: determining research priorities, contributing to research design, establishing a community advisory board (CAB), supporting recruitment efforts, participating in data analysis, and engaging in dissemination activities [28].

## 3. Results

### 3.1. Study Characteristics

**Figure 1:**
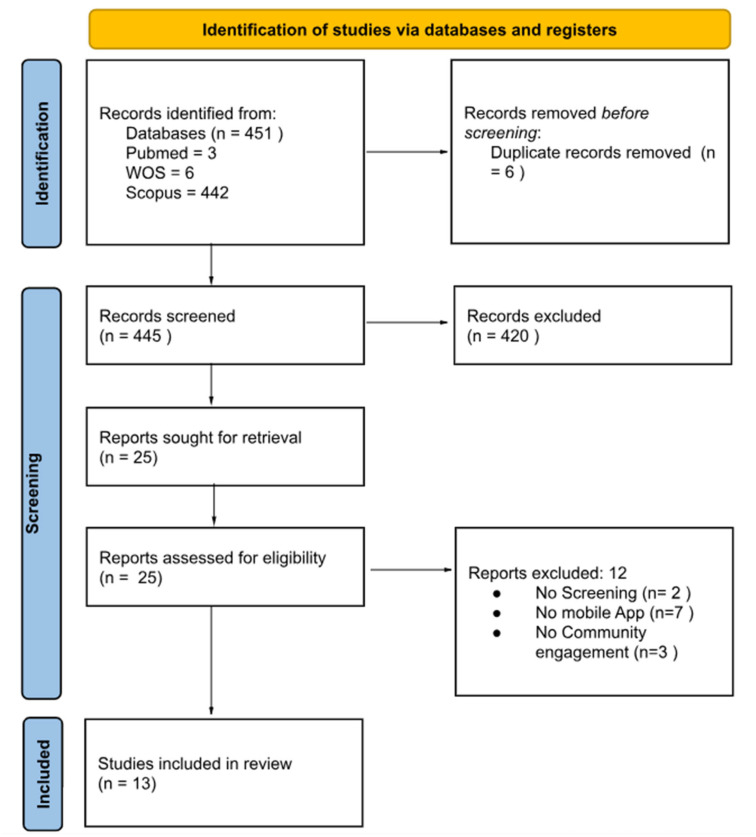
PRISMA flow chart detailing the records identified and screened, the number of full-text articles retrieved, and the reasons for final exclusion.

A total of 445 articles were identified. Twenty-five papers were assessed for inclusion criteria after full-text retrieval. The full selection process is illustrated in the PRISMA flow diagram (Figure 1).

The thirteen included papers could be linked to four apps created through community engagement: the wMammogram [29,30], a virtual clinician (VC) called Meet ALEX [31,32,33,34,35,36,37], the ColorApp [38,39], and the mMammogram [40,41]. The characteristics of the included studies are summarized in Table 1.

Of the studies that met the criteria to be included, the oldest was published in 2017 [41].

One randomized-controlled trial (RCT) [29] and one cross-sectional study [30] related to the wMammogram app were retrieved. Meet ALEX was described in seven papers published between 2019 and 2022, with the following study designs: one abstract meeting [32], one quasi-experimental study [34], one randomized trial [35], three cross-sectional studies [31,33,37], and one bibliometric review [36]. Papers related to the ColorApp have a quasi-experimental design [38] and a cross-sectional study design [39]. The mMammogram app was described in one RCT [41] and one cross-sectional study [40] published in 2017 and 2018, respectively.

Each mobile application was developed for a single cancer screening program without the availability of mHealth apps covering multiple cancer screening at once.

### 3.2. Performance of mHealth Apps

The use of the wMammogram app showed a higher percentage of women receiving mammograms, a greater intention to receive it in the future, better BC and BC screening knowledge, and a willingness to recommend the intervention. During the development of the Meet ALEX application, four community perceptions were investigated to determine its usability: “authority”, i.e., the ability of the virtual clinician to have a formal position or appearance; “expertise”, i.e., participants’ perception of the virtual clinician’s medical skills; “friendliness”; and “trustworthiness”. Participants reported their willingness to receive and share information about CRC screening. Women participating in the mMammogram app development stated that using the app increased their “awareness, positive attitude, and knowledge on breast cancer and breast cancer screening methods” and their “understanding of the importance of regular mammography”, and it reduced their “anxiety about mammography, resulting in promoting their interests in screening participation.” Overall, the ColorApp app showed an improvement in knowledge scores, but not in attitudes. Participants reported good usability of the mobile application.

### 3.3. Phases of Community Participation

Main phases of Community Participation are summarized in Table 2. None of the studies described the involvement of the community in setting priorities. Therefore, it is unclear whether the increased cancer incidence or low adherence to screening in the target population were issues perceived by the community. For the “research design” phase, all target communities were involved. In developing the wMammogram application, American Indian women helped in “identifying barriers and mobile phone usage patterns, creating motivators, tailoring message web app content, and developing appropriate behavioral motivators and triggers”. American Black men from the community helped to identify the needs to be addressed by the Meet ALEX application and contributed to the development and evaluation of the prototype. Korean American immigrant women were involved in mMammogram development, helping in research design by suggesting how to improve the efficacy of the program. ColorApp was developed within a Community-Based Intervention program called KOSPEN. Three individuals from the intended user group (Malaysian men and women aged 50 years or older) were involved in meetings to explore the information needs related to CRC screening, health education, and promotion in Malaysia, to tailor the messages and to improve the usability of the app. Regarding the establishment of a CAB, one was created in the cases of wMammogram, Meet ALEX, and mMammogram applications. It was not possible for the researchers to verify the existence of a CAB for the development of the ColorApp application, as much of the material related to the KOSPEN program is not accessible; regardless, the documents available never mention a CAB. Community participation in the “recruitment” phase was present in all included studies. In the development of the mMammogram, the CAB was involved in the data analysis phase, as it was reported to help in the interpretation of preliminary results. The two applications developed using the CBPR framework for community involvement included participation in the “dissemination” phase of the results as one of the tasks assigned to the CAB. In the papers describing the development of the mMammogram and wMammogram apps, the women who participated in the research are acknowledged. In addition, the community involved in the KOSPEN program is mentioned in the acknowledgements within the articles related to the development of the ColorApp app.

## 4. Discussion

In this scoping review, we explored the available literature regarding the promotion of cancer screening to increase program adherence through an mHealth app specifically designed and implemented through a community engagement approach. Our findings suggest that the body of evidence is still growing, with a prevalence of studies focusing on CRC and usually among minorities. The categorization of the app development process into phases of community engagement showed that the research design, the establishment of a CAB, and the recruitment phase were the most represented, whereas the priority setting phase was never shared with the target community; data analysis and dissemination were occasionally performed.

Exploring the key factors that determine the success of screening programs, evidence suggests that nurturing trust among the target population towards both their healthcare providers [42] and the institutions, as underlined by the WHO [43], as well as collaboration with local civil society organizations, are extremely important elements [8].

The concept of co-creation, which involves collaboration among individuals with diverse roles and positions to achieve shared objectives, is gaining recognition as a valuable method for engaging partners and communities in research [44].

In some studies, the target population is involved in specific phases of a screening program implementation, but without adhering to a specific engagement framework [45]. Thus, the results, whether positive or negative, cannot be attributed to the study methodology and are not comparable with other similar studies. This makes such studies compelling, but their low external validity makes them difficult to replicate. It is important to refer to standardized community engagement methods for the co-creation of applications to promote cancer screening, to better delineate the differences between the traditional and the community engagement approaches: this will allow the development of secondary studies that produce syntheses of evidence, which are currently very scarce on this topic [46,47]. Among the four studies included, two app developments adopted a CBPR approach, which focuses on developing a collaborative alliance between the research team and the population of interest [48]. Of note, in both studies, the community was explicitly involved in a greater number of stages than in the other two adopted frameworks, suggesting that the CBPR is an effective approach in reducing inequities [49].

No community engagement application has been developed for CC screening. This is surprising for two reasons: first, the target population for this screening is considerably younger, especially with respect to CRC, and one would expect them to be the most likely to use mHealth applications. Second, although CC has a relatively low global burden, it is the most common cancer among women in certain limited communities, which may justify the development of community-based programs [2].

Improving cancer screening and early detection is a gradual process that may take years to show results, so immediate changes are unlikely to be noticeable or easy to measure [50]. Only two articles assessed screening test performance at six-month follow-up as an outcome [29,40]. Thus, the published data are not sufficient to determine whether the development of applications through community engagement produces a significantly different outcome than other types of intervention to promote adherence to screening.

It has been shown that the barriers and facilitators to adherence to screening programs are largely cross-cutting, and that working with the community as a whole, rather than just the specific target population, helps to create a social culture of trust in screening programs, ultimately increasing participation [51]. Nonetheless, in line with previous publications [52], all the included studies focused on a specific screening (i.e., CRC or BC), without taking advantage of the opportunity to involve populations eligible for multiple screenings based on age group. Allocating resources for community engagement with specific populations and developing applications for each individual screening may prove too demanding and not sustainable in all countries. Moreover, another aspect that might hinder the understanding of barriers to adherence is the necessity for qualitative methodologies to be integrated into the app development process. Unlike quantitative methods, qualitative approaches allow us to explore the community beliefs and stigmas regarding cancer screening, and ultimately address them [52]. According to a scoping review by Ravaghi and colleagues, mixed-methods approaches are increasingly being used, as they have proven to be more inclusive and effective in engaging hard-to-reach communities [53]. Lastly, as far as patient needs and resources are concerned, both quantitative and qualitative methodologies should be employed, as evidence suggests that addressing these in every step of the program development might increase motivation [54] and readiness to change [55], a crucial element to engagement.

Further research should focus on filling the gap in the literature regarding CC screening programs overall and, more specifically, how to efficiently adopt and incorporate community engagement frameworks, such as CBPR, in implementing a CC screening program, with or without mHealth. Another noticeable gap to be addressed is the involvement of the community in the “priority setting” phase; as mentioned, exploring the community needs and resources in every step, but especially in the earliest stages, might greatly influence the actual program outcomes in terms of adherence and engagement. Moreover, studies exploring the long-term effects of mHealth interventions in terms of, for example, direct and/or indirect costs, are required to ensure the robustness and the continuous quality improvement of programs.

## 5. Conclusions

Cancer screening programs implemented through mHealth apps conceived using community engagement approaches have proven to be an effective way of promoting adherence to screening, as they yield more personalized, tailored interventions that reflect the target community characteristics. CRC screening is the most studied, whereas CC is the most understudied; furthermore, studies that rely on established frameworks (i.e., CBPR) in the literature make their methodology more comparable and replicable in different contexts, facilitating the spread of good practices, especially when the development of mHealth tools, like apps, is involved.

## Figures and Tables

**Table 1:** Characteristics of mHealth apps described in the included studies.

App Name	Screening	Country	Population	App Features	Measured Outcomes	Measurement Tools
wMammogram	BC	USA	American Indian women aged 40 to 70	Educational messages about BC and BC screening; information about financial support and resources; GPS navigation to nearby clinics; ability to send reminders and personalize frequency of educational messages.	BC screening adherence, attitude, knowledge, and willingness to recommend the intervention	Focus groups, questionnaires
Meet ALEX	CRC	USA	Rural adults aged 50 to 73	Virtual clinician tailored to reflect the demographic background of the patient, delivering education about CRC cancer and instructions for using the FIT.	App usability, CRC screening knowledge and attitude	Focus groups, interviews, questionnaires
mMammogram	BC	USA	Korean immigrant women aged 40 to 79	Messages using culture-specific emoticons, graphs, images, pictures, and videos in the Korean language. Bilingual health navigation services, information about financial support and resources, and a link to a website containing a list of area clinics.	BC screening adherence and knowledge, app usability	Questionnaires, interviews
ColorApp	CRC	Malaysia	Malaysian males and females, 50 years and older	Educational messages and videos on CRC and CRC screening in Malaysia; a health calculator to suggest the need for screening; tracking of body weight, blood pressure, glucose, and cholesterol levels; support for tobacco cessation.	App usability, CRC screening knowledge and attitude	Questionnaires

CRC = colorectal cancer; BC = breast cancer; FIT = fecal immunochemical test.

**Table 2:** A summary of the presence of the phases of community participation in the retrieved articles. The Phases of Community Engagement is adopted in each app development process.

App Name	CE Framework	Phases of Community Engagement					
		Priority Setting	Research Design	CAB	Recruitment	Data Analysis	Dissemination
wMammogram	CBPR		X	X	X		X
Meet ALEX	UCD		X	X	X		
mMammogram	CBPR		X	X	X	X	X
ColorApp	CBI		X		X		

CE = Community Engagement; CBPR = Community-Based Participatory Research; UCD = user-centered design; CBI = Community-Based Intervention; CAB = community advisory board; X = Phases of Engagement used for the development of specific Apps.

## Data Availability

No new data were created or analyzed in this study. Data sharing is not applicable to this article.
